# Identification of host protein ENO1 (alpha-enolase) interacting with *Cryptosporidium parvum* sporozoite surface protein, Cpgp40

**DOI:** 10.1186/s13071-024-06233-5

**Published:** 2024-03-19

**Authors:** Yuexin Wang, Na Li, Guanda Liang, Luyang Wang, Xiaotian Zhang, Zhaohui Cui, Xiaoying Li, Sumei Zhang, Longxian Zhang

**Affiliations:** 1 College of Veterinary Medicine, Henan Agricultural University, Zhengzhou, 450046 Henan People’s Republic of China; 2 International Joint Research Laboratory for Zoonotic Diseases of Henan, Zhengzhou, 450046 Henan People’s Republic of China; 3 Key Laboratory of Quality and Safety Control of Poultry Products, Ministry of Agriculture and Rural Affairs, Zhengzhou, 450046 Henan People’s Republic of China

**Keywords:** *Cryptosporidium parvum*, ENO1, Cpgp40, GST pull-down, Interaction

## Abstract

**Background:**

*Cryptosporidium parvum* is an apicomplexan zoonotic parasite causing the diarrheal illness cryptosporidiosis in humans and animals. To invade the host intestinal epithelial cells, parasitic proteins expressed on the surface of sporozoites interact with host cells to facilitate the formation of parasitophorous vacuole for the parasite to reside and develop. The gp40 of *C. parvum*, named Cpgp40 and located on the surface of sporozoites, was proven to participate in the process of host cell invasion.

**Methods:**

We utilized the purified Cpgp40 as a bait to obtain host cell proteins interacting with Cpgp40 through the glutathione *S*-transferase (GST) pull-down method. In vitro analysis, through bimolecular fluorescence complementation assay (BiFC) and coimmunoprecipitation (Co-IP), confirmed the solid interaction between Cpgp40 and ENO1. In addition, by using protein mutation and parasite infection rate analysis, it was demonstrated that ENO1 plays an important role in the *C. parvum* invasion of HCT-8 cells.

**Results:**

To illustrate the functional activity of Cpgp40 interacting with host cells, we identified the alpha-enolase protein (ENO1) from HCT-8 cells, which showed direct interaction with Cpgp40. The mRNA level of *ENO1* gene was significantly decreased at 3 and 24 h after *C. parvum* infection. Antibodies and siRNA specific to ENO1 showed the ability to neutralize *C. parvum* infection in vitro, which indicated the participation of ENO1 during the parasite invasion of HCT-8 cells. In addition, we further demonstrated that ENO1 protein was involved in the regulation of cytoplasmic matrix of HCT-8 cells during *C. parvum* invasion. Functional study of the protein mutation illustrated that ENO1 was also required for the endogenous development of *C. parvum*.

**Conclusions:**

In this study, we utilized the purified Cpgp40 as a bait to obtain host cell proteins ENO1 interacting with Cpgp40. Functional studies illustrated that the host cell protein ENO1 was involved in the regulation of tight junction and adherent junction proteins during *C. parvum* invasion and was required for endogenous development of *C. parvum*.

**Graphical Abstract:**

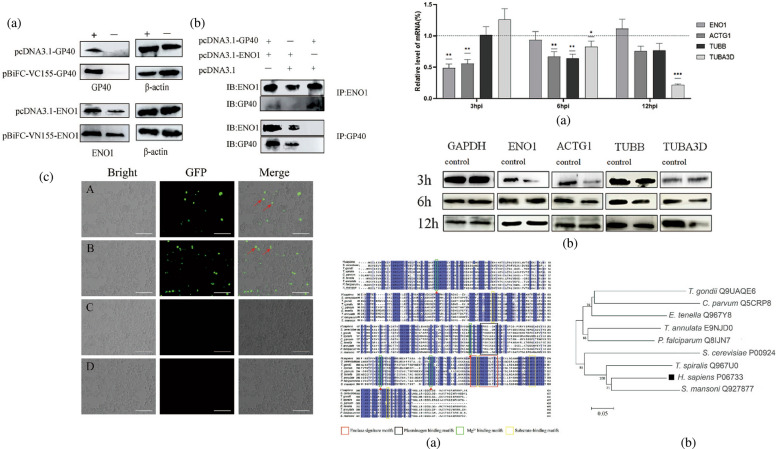

**Supplementary Information:**

The online version contains supplementary material available at 10.1186/s13071-024-06233-5.

## Background

*Cryptosporidium* is an important zoonotic parasite that can infect a wide range of hosts, causing diarrheal diseases worldwide for which no drugs are available to treat those severely infected cases [for example, human immunodeficiency virus (HIV) patients] [1, 2]. Cryptosporidiosis could be life-threatening in young infants as well as people who are severely malnourished or immunocompromised [3, 4]. More importantly, in addition to causing diarrhea, human cryptosporidiosis are frequently associated with weight loss, growth stunting, cognitive impairment in children, and it has been estimated that *Cryptosporidium* cause over 13 million disability-adjusted life years (DALYs) annually[5–8].

To date, a total of 44 valid *Cryptosporidium* species have been reported, and *C. parvum* is responsible for the majority of zoonotic infections [9, 10]. It is clear that *C. parvum* targets host intestinal epithelial cells for an intracellular but extracytoplasmic localization [11]. However, mechanisms of *C. parvum* invasion into host cells are not fully understood. Studies on motility, adhesion, and invasion of *C. parvum* have identified several parasitic proteins and virulence factors involved in the parasite interaction with host cells, including proteins localized on the surface of sporozoites (i.e. gp40/15) and proteins derived from secretary organelles (i.e. rhoptry bulk proteins) [12–17]. It is believed that sporozoite surface proteins directly interact with host cells, whereas secretary proteins are released or injected to interact with either host cell membrane proteins or cytoplasmic proteins [18, 19]. The gp40/15 of *C. parvum*, referred to as Cpgp40/15, is encoded by the *gp60* (or *gp40/15*) gene of *C. parvum* [17, 20]. The *gp60* gene is considered to be the virulence determining region of *Cryptosporidium*, which has been widely used in the genotyping of *Cryptosporidium* [21]. In early studies, Cpgp40/15 was reported as a precursor protein, which can be proteolytically cleaved into glycoproteins Cpgp40 and Cpgp15 by both human and parasite-derived furin and furin-like protease [22]. Characteristics of the gp40 protein include an N-terminal signal peptide, a polyserine-domain, multiple *O*-glycosylation sites, and single N-glycosylation site [23]. Unlike Cpgp15, which has a glycosylphosphatidyl inositol (GPI) moiety for anchoring sporozoites membrane, Cpgp40 does not have any features for membrane localization [17]. However, immunofluorescence staining of Cpgp40 and Cpgp15 observed that the two glycoproteins colocalized on the surface of sporozoites [24]. Antibody neutralization of Cpgp40 clearly demonstrated the significant decreasing of parasite infection into intestinal epithelial cells, indicating that Cpgp40 is involved in the *C. parvum* adhesion and invasion into host cells [17, 25]. Moreover, in vitro binding assay illustrated that Cpgp40 binds to intestinal epithelial cells in a dose-dependent and saturable manner [17], suggesting the possibility of Cpgp40 interacting with a host cell receptor.

In the present study, we utilize the purified Cpgp40 as a bait to obtain host cell proteins interacting with Cpgp40 through the glutathione *S*-transferase (GST) pull-down method. Bioinformatics analysis and differential analysis of protein expression narrowed it the alpha-enolase (ENO1) as the key host cell protein that has direct interaction with Cpgp40. In vitro analysis, through bimolecular fluorescence complementation assay (BiFC) and coimmunoprecipitation (Co-IP), confirmed the solid interaction between Cpgp40 and ENO1, demonstrating that ENO1 was the host cell protein directly interacting with Cpgp40. Subsequent functional studies illustrated that the host cell protein ENO1 was involved in the regulation of tight junction (TJ) and adherent junction (AJ) proteins during *C. parvum* invasion. Meanwhile, by using protein mutation and parasite infection rate analysis, it was demonstrated that ENO1 was required for endogenous development of *C. parvum*.

## Methods

### Parasites and cell lines

Fresh oocysts of *C. parvum* with a subtype IIdA19G1 at *gp60* locus were collected from neonatal dairy calves with clinical symptoms of diarrhea. *C. parvum* oocysts used in experiments were < 3 months old and purified by Sheather’s sugar flotation and cesium chloride density gradient centrifugation. Finally, they are stored at 4°C in phosphate-buffered saline solution (PBS). Prior to use, oocysts were centrifuged at 5000×*g* for 10 min, treated with 2.5% Clorox on ice for10 min and washed three times with PBS. Free *C. parvum* sporozoites were obtained by incubation in PBS containing 0.25% trypsin and 0.5% taurodeoxycholic acid at 37 °C for 0.5 h, followed by washing three times with PBS.

Human ileocecal adenocarcinoma (HCT-8) cells and human embryonic kidney 293 cell (HEK293) (Chinese Academy of Sciences) were cultured and maintained in RPMI 1640 and Dulbecco’s modified Eagle medium (DMEM) media (Hyclone, USA) containing 10% fetal bovine serum (FBS), 100 U/ml penicillin, and 0.1 mg/ml streptomycin. The proportion of infection used in vitro *Cryptosporidium* infection cell models is 5:1 between oocysts and HCT-8 cells.

### Recombinant GST-Cpgp40 expression, purification, and polyclonal antibodies preparation

A 588-bp fragment encoding the full-length protein of Cpgp40 was inserted into the expression vector pGEX-4T-1 (Novagen, Madison, WI), resulting in a construct that expresses a 190 amino acid fusion protein. Recombinant vectors pGEX-4T-1-Cpgp40 and recombinant proteins GST-Cpgp40 are preserved in our laboratory. Anti-Cpgp40 polyclonal antibodies was obtained in pathogen-free rabbits by Sangon Biotech (Shanghai, China) and stored at −20 °C. Its titer and specificity were evaluated as described [25].

### Identification of potential Cpgp40-interacting proteins

2–5 × 10^7^ HCT-8 cells was collected by centrifugation at 500*g* and 4 °C for 5 min and washed three times with phosphate-buffered saline (PBS) at 500*g* and 4 °C for 5 min. HCT-8 cell membrane proteins (1.5 mg/ml) were extracted according to Minute™ plasma membrane protein isolation and cell fractionation kit (Invent Biotechnologies, UK). Purified GST-Cpgp40 (or GST as control) fusion proteins were immobilized with Glutathione Sepharose 4B beads (GE Healthcare, USA) at 4 °C for 2 h after beats being balanced. Then, GST pull-down assays was conducted as described previously [26]. The samples were sent to Genecreate Biological Engineering (Wuhan, China) for identification (Additional files 1 and 6).

### The key host cell proteins expression in various developmental stages of *C. parvum*

The relative expression levels of the *ENO1*, *ACTG1*, *tubulin beta chain* (*TUBB*), and *TUBA3D* genes in intracellular parasites in HCT-8 cultures at 0–12 h (3, 6, and 12 h postinfection) was evaluated by quantitative PCR (qPCR) (killed parasites were used as control). The expression of the *GAPDH* gene was determined in parallel for data normalization. The total RNA (0.3 mg/ml) was isolated from infected HCT-8 cultures using TRIzol (Invitrogen, USA). Afterwards, cDNA was synthesized from the RNA using ReverTra Ace^®^qPCR RT Master Mix kit (Toyobo, Japan). The qPCR was performed in a 20 μL reaction containing 0.1 mM primers, 2 µL of cDNA, and 10 µL of SYBR Green PCR Mix (Toyobo, Japan) with the following cycling conditions: 95 °C for 30s, 40 cycles of 95 °C for 10 s, 60 °C for 10 s, and 72 °C for 15 s. The qPCR primer sequences of these genes used in this study are presented in Additional file 2: Table S3. The relative expression quantities of each gene were calculated by using the comparative Ct method (2^−ΔΔCt^).

### Plasmid construction

The expression vector pcDNA3.1 and pCAGGS (Invitrogen, USA) were used to generate prokaryotic and mammalian expression constructs carrying the full-length Cpgp40 or human ENO1 (GenBank accession no. M14328.1). The full-length Cpgp40 coding sequence was PCR amplified from pGEX-4T-1-Cpgp40 using primers (Additional file 2: Table S1). And coding sequence of ENO1 was PCR amplified from cDNA of HCT-8 cell using corresponding primers (Additional file 2: Table S1). These two sequences were cloned into pcDNA3.1 between *EcoR I* and *Xho I* sites with adding the kozak sequence to the upstream sequence.

The plasmids for bimolecular fluorescence complementation assay (pBiFC-VN155 and pBiFC-VC155, Miaoling Bio, China) were used to express the fusion protein of N-fragment and C-fragment. The fragments of Cpgp40 gene and ENO1 gene were cloned into these two vectors respectively between *EcoRI* and *XhoI* sites, with adding the kozak sequence as well. All the primers and plasmids constructed in this study are listed in Additional file 2: Tables S1 and S2.

### Bimolecular fluorescence complementation

HEK239 cells were seed into six-well plates and grown to ∼70–80% confluency for transfection. To examine the interactions between Cpgp40 and ENO1, plasmids pBiFC-VC155-HA-Cpgp40 and pBiFC-VN155-ENO1 were cotransfected at the ratio of 1:1 into cells using Lipofectamine 2000. Plasmids pBiFC-bfosVC155 and pBiFC-bjunVN155 were cotransfected as positive control and pBiFC-bfosVC155 (delta ZIP) and pBiFC-bjunVN155 as negative control. The process involves the fusion of two nonfluorescent fragments of a fluorescent protein to the target proteins under investigation. These fragments, when brought into close proximity due to the interaction of the fused proteins, reconstitute a functional fluorophore, leading to the emission of a fluorescent signal. The cells were then incubated at 37 °C (with 5% CO_2_) for 24 h and subsequently imaged using the fluorescent microscope (Olympus, Japan). The bimolecular fluorescence complementation (BiFC) fluorescence was excited at 543 nm and detected within a range from 580 to 680 nm.

### Coimmunoprecipitation

Plasmids (1 mg/ml) expressing HA-Cpgp40 (pcDNA3.1-HA-Cpgp40) and ENO1 (pcDNA3.1-ENO1) were mixed at the ratio of 1:1 and transfected into HEK293 cells in three-well plates using Lipofectamine 2000 (Invitrogen, USA) according to the manufacturer’s instructions. As controls, cells were also transfected with plasmids pcDNA3.1-ENO1 or pcDNA3.1-HA-Cpgp40 under the same conditions. At 48 h post-transfection, cells were lysed in weak RIPA lysis buffer (Solarbio, China) containing 1 mM phenylmethylsulfonyl fluoride (Solarbio, China) and protease inhibitor cocktail (Solarbio, China). The cell lysates were incubated with mouse anti-ENO1 antibody or anti-HA antibody (Abcam, UK) at 4 °C overnight, followed by antibody capture by Protein G agarose beads (Santa Cruz Biotechnology, CA) at 4 °C for 3 h. The agarose beads were then extensively washed with weak RIPA lysis buffer containing 1 mM phenylmethylsulfonyl fluoride and protease inhibitor cocktail and PBS containing 1 mM phenylmethylsulfonyl fluoride and protease inhibitor cocktail. Proteins bound to agarose beads were solubilized in 1× SDS-loading buffer, boiled at 100 °C for 10 min, and examined by immunoblotting analysis.

### Immunoblotting

The protein samples (1.5 mg/ml) were separated on 10% SDS-PAGE gels and transferred electrophoretically to polyvinylidene fluoride (PVDF) membranes (Millipore, USA). The membranes were first blocked with 1% bovine serum albumin for 2 h at room temperature and then incubated at 4 °C overnight with corresponding primary antibody. Primary antibodies were detected by horseradish peroxidase (HRP)-conjugated secondary antibodies followed by enhanced chemiluminescence (ECL) (Millipore, USA) to quantify HRP levels.

### Short-interfering (si)RNA

Three siRNAs targeting ENO1 mRNAs and one scramble siRNA (nontargeting control siRNA, si-NC, negative control) were designed by the Ribo Biotech (Guangzhou, China). HCT-8 cells were grown to 60–70% confluency in 12-well cell culture plates and transfected with siRNAs (30 nM, 50 nM, and 100 nM) using ribo FECT™ CP transfection reagent (Ribo Biotech, China). The extent of inhibition was determined by qPCR and western blot assays of ENO1 expression at 24 h post-transfection. The siRNA sequence that caused the greatest inhibition of ENO1 expression was GCTGCTGAAGACTGCTATT, and this inhibitory effect was observed at a transfection concentration of 100 nM both for si-ENO1 and si-NC (negative control).

### Effects of ENO1 on parasite infection

The effect of polyclonal antibodies against ENO1 on *C. parvum* infection of HCT-8 cells was examined using an in vitro neutralization assay. HCT-8 cell monolayers in 24-well plates were preincubated with anti-ENO1 antibody for 1 h prior to the addition of *C. parvum* oocysts. The final concentration of antibody was 10 μg/well. HCT-8 cell were also seed in 24-well plates for transfection of overexpression vector pcDNA3.1-ENO1 and si-ENO1 RNA. The negative control cells that were infected with heat-killed parasites was set in parallel for data normalization. Fresh sporozoites were incubated with HCT-8 cells for 1 h at 37 °C (killed parasites were used as control) and then removed free parasites by an exchange of culture medium. The cells were washed with RNase-free PBS three times and harvested at 3 h and 18 h after infection for the isolation of total RNA and detection of parasite load by qRT-PCR as described below. Human 18s gene and *C. parvum* 18s gene primes are present in Additional file 2: Table S3.

### Preliminary biological function analysis of human ENO1 in *C. parvum* invasion

HCT-8 cells were seed into 12-well plates and 6-well plates at ~ 70% confluence as described as above. Overexpression vector pcDNA3.1-ENO1 and si-ENO1 RNA were transinfected for 24 h. After *C. parvum* oocysts were added for 24 h (killed parasites were used as control), total cDNA and proteins were obtained. Human TJ and AJ protein genes were amplified with gene‐specific primers utilizing human GAPDH, respectively, as internal controls. The primer sequence of the genes used are present Additional file 2: Table S3. Levels of TJ/AJ proteins were immunodetected using specific antibodies (Proteintech, China) and visualized by enhanced chemiluminescence reagents.

### Alignment of amino acid sequence and phylogenetic analysis

Human ENO1 DNA and protein sequences was analyzed by the BLAST tool (http://blast.ncbi.nlm.nih.gov/Blast.cgi) for homology search. And they were compared with enolase sequences from a wide range of fungus and parasites species including *Saccharomyces cerevisiae*, *Toxoplasma gondii*, *Trichinella spiralis*, *Cryptosporidium parvum*, *Eimeria tenella*, *Theileria annulate*, *Plasmodium falciparum*, and *Schistosoma mansoni*. Additionally, the conserved motifs, signal peptides, transmembrane regions and glycosyl-phosphatidyl anchor sites of the enolase sequence were analyzed by the online resources, including the National Center for Biotechnology Information (NCBI) conserved domains (http://www.ncbi.nlm.nih.gov/Structure/cdd/wrpsb.cgi), SignalP 4.1 Server (http://www.cbs.dtu.dk/services/SignalP), TMHMM Server version 2.0 (http://www.cbs.dtu.dk/services/TMHMM-2.0/) and Clustal Omega (http://www.clustal.org/omega/). The final result was present using Jalview, version 2.11 (http://www.jalview.org/). Phylogenetic trees were constructed using neighbor-joining methods (Kimura two-parameter model) using MEGA, version 7.0 (http://www.megasoftware.net/), and a bootstrap analysis with 1,000 replicates was used to assess tree reliability.

### Effect of truncated ENO1 protein on the *C. parvum* invasion of HCT-8 cells

Based on the analysis of human ENO1 amino acid sequences, we mutate the metal ion binding site (S^37^, D^245^, E^293^, and G^317^), which play important role in glycolysis to alanine with short side chains. Four pairs of mutant primers are designed (Additional file 2: Table S1) to construct a eukaryotic expression vector pCAGGS-ENO1-M for mutant proteins. Using the pCAGGS-HA-ENO1 as a template, the above primers are used for amplification separately(95 °C for 30 s; 95 °C for 15 s and 62 ℃ for 15 s, 30 cycles; and 72 ℃, 60 s/5 min). Four PCR amplification products were digested by Dpn I to remove methylated template plasmid and then ligated using Mut Express^®^ MultiS Fast Mutagenesis Kit V2 (Vazyme, China).

HCT-8 cells were seed into 12-well plates at ~70% confluence as described as above. Eukaryotic expression vector pCAGGS-ENO1 and pCAGGS-ENO1 were transfected into HCT-8 cells in three replicates. After 24 h, *C. parvum* oocysts were added into the culture and the extent of *C. parvum* invasion was determined by at least two replicate qRT-PCRs for each sample at 3 h and 18 h postinvasion.

### Statistical analysis

Data are presented as means ± standard error of the mean (SEM) of different independent experiments. Differences between controls versus treated groups were analyzed using one‐way analysis of variance with Tukey’s test or Student’s *t* test. All statistical analyses were processed using GraphPad PRISM 6.07 software (San Diego, CA, USA) or IBM SPSS software (Release 13.0 standard version; SPSS Inc., Chicago, IL, USA). Values of *P* < 0.05 were considered statistically significant.

## Results

### Identification of host proteins interacting with Cpgp40

To screen and identify host proteins that interact with Cpgp40, a high throughput protein–protein interaction assay, GST pull-down, was performed. According to the method described previously, cell membrane lysate derived from HCT-8 cells was incubated with the recombinant GST-Cpgp40 or GST protein and analyzed on an SDS-PAGE gel by silver staining (Additional file 3: Fig. S1). The mass spectrometry (MS) results were shown in Additional file 4: Fig. S2 and Additional file 5: Table S4. Bioinformatics analysis of these proteins indicated that most of them are distributed on cell membrane and cytoskeleton and are closely related to pathogen invasion and cell function. Since the invasion of *Cryptosporidium* is closely related to cell membrane and cytoskeleton proteins, we selected proteins targeted at cell membrane and cytoskeleton to be included in further analysis.

### The expression profile of key host cell proteins upon *C. parvum* infection

**Fig. 1:**
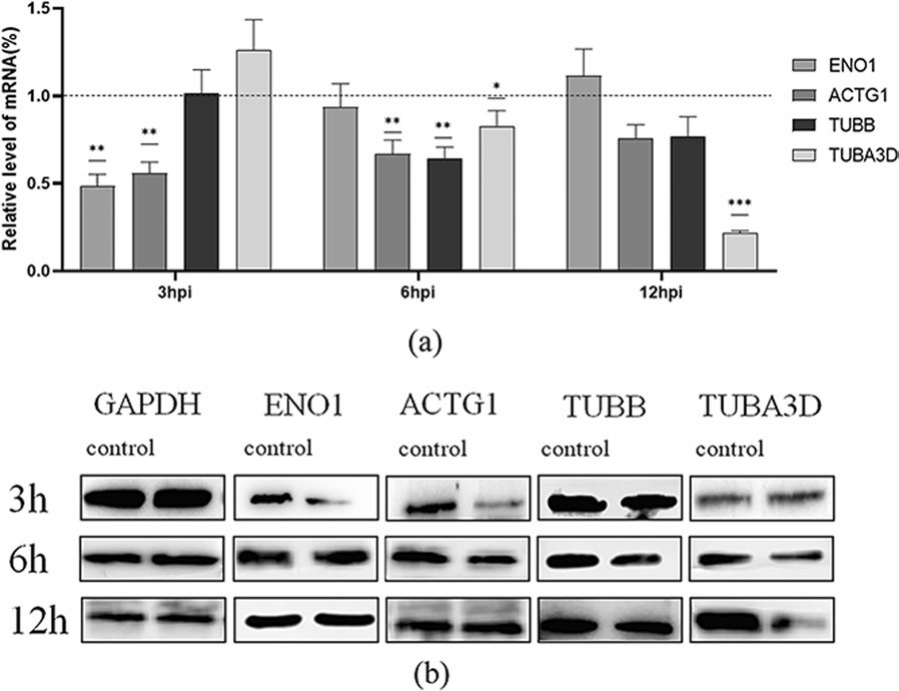
Relative mRNA (**a**) and protein (**b**) level of key host cell proteins (ENO1, ACTG1, TUBB, and TUBA3D) in HCT-8 cell culture. The qPCR result is displayed after normalizing the Ct values with data from the GAPDH gene of HCT-8 cells. Data shown are mean ± SD from three replicate assays

To detect several human genes expression (*ENO1*, *ACTG1*, *TUBB*, and *TUBA3D*) upon *C. parvum* infection, we selected three time points (3, 6, and 12 h) for mRNA and protein expression level detection. The qPCR result showed that these genes were downregulated upon *C. parvum* infection at 3 h, 6 h and 12 h (Fig. 1). We observed that ENO1 gene expressed in HCT-8 cells was downregulated upon *C. parvum* infection at 3h which is the early stage of infection(*P* = 0.00169 < 0.01). The ACTG1 gene was downregulated at 3h (*P* = 0.0009 < 0.1) and 6h (*P* = 0.0019 < 0.01) significantly. TUBB was downregulated at 6 h (*P* = 0.0017 < 0.01), and TUBA3D gene was downregulated at 6 h (*P* = 0.0268 < 0.05) and 12 h (*P* < 0.0001) (Fig. 1).

### Direct interaction between Cpgp40 and ENO1

**Fig. 2:**
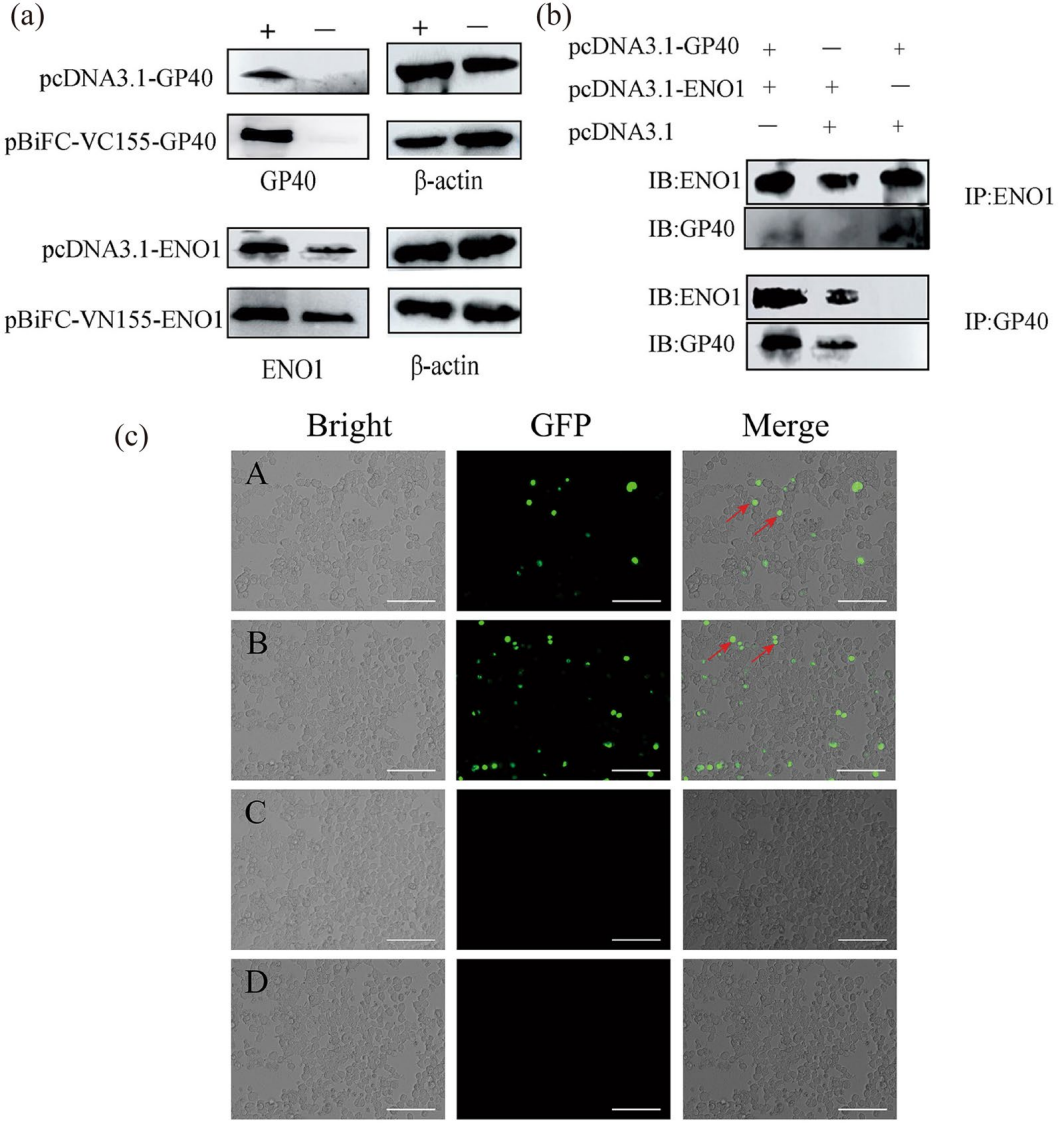
Confirmation of the interactions between Cpgp40 and host protein ENO1. **a** Verification of recombinant protein expression in HEK293T cells. **b** Interactions between ENO1 and Cpgp40 in vivo as determined by coimmunoprecipitation. **c** Bimolecular fluorescence complementation (BiFC) assessing the interactions between ENO1 and Cpgp40 in live mammalian cells. A: positive control: pbJun-VN155 + pbFos-vc155. B: pBiFC-VC155-GP40 + pBiFC-VN155-ENO1. C: negative control: pbJun-VN155 + pbFos-vc155(delta ZIP). D: blank. (scale bar: 100 μm). The red arrow points to the fluorescent signal

To directly observe the interactions between Cpgp40 and ENO1 in live mammalian cells, we conducted bimolecular fluorescence complementation assay (BiFC). Mature Cpgp40 fused to VC155 and ENO1 fused to VN155 were coexpressed in HEK293 cells (Fig. 2a). We also coexpressed bfosVC155 and bjunVN155 as a positive control. The result indicated that cells cotransfected with Cpgp40-VC155 and ENO1-VN155 showed punctate green fluorescence (BiFC signal) in HEK293 cells, which was similar with the positive control. No green signal in negative control cells cotransfected with bfos-VC155 (delta ZIP) and pbJun-VN155 were observed, as well as in blank group (Fig. 2c).

To further confirm these interactions in mammalian cells, we cotransfected plasmids expressing ENO1 and HA-Cpgp40 into HEK293T cells for immunoprecipitation assays (Co-IP). ENO1 and HA-Cpgp40 were divide into two groups and immunoprecipitated with an anti-ENO1 antibody and anti-HA antibody, respectively. The bound ENO1 or Cpgp40 was detected by an anti-ENO1 or HA antibody. The results showed that human ENO1 was able to be coprecipitated by HA-Cpgp40 (Fig. 2b). These results suggest that these two proteins can interact with each other in mammalian cells.

### The presence of antibody specific to ENO1 and si-ENO1 could reduce *C. parvum* infection

**Fig. 3:**
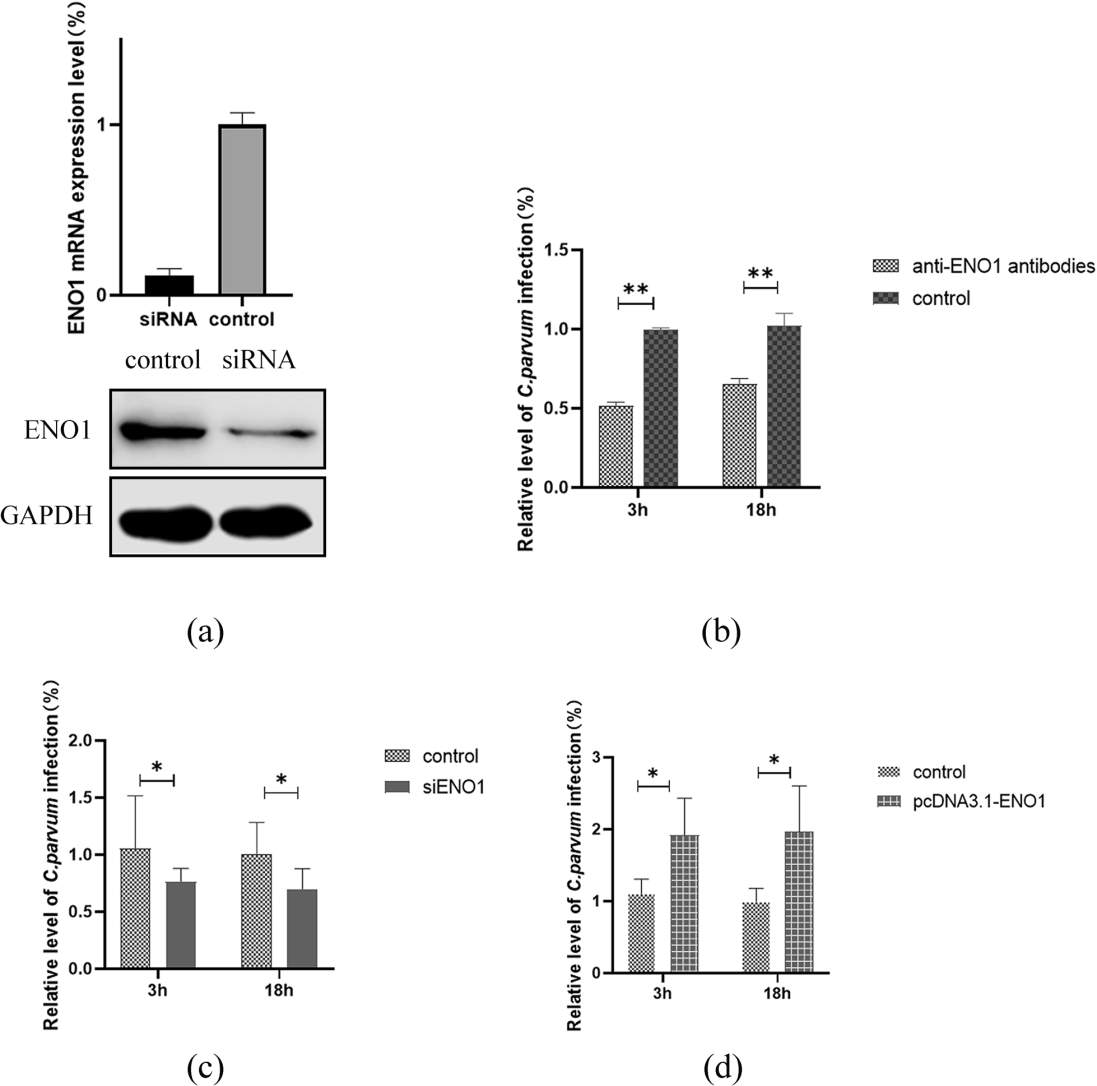
The silent efficiency detection of siRNA and inhibition of *Cryptosporidium parvum* invasion of HCT-8 cells by monoclonal ENO1 antibodies, siRNA, and overexpression of ENO1. **a** The silent efficiency detection of siRNA. GAPDH was used as an internal reference protein. **b**–**d** Inhibition of *C. parvum* invasion of HCT-8 cells. Human SSU rRNA gene was used as an internal control. Values are mean ± SEM. control: **b** PBS; **c** si-NC; **d** pcDNA3.1 blank vector

We designed siRNA that can silence ENO1 gene expression and transinfected it into HCT-8 cells to test the effect of reduced ENO1 expression on *C. parvum* infection. In comparison with control, the level of ENO1 mRNA was reduced by about 70–80%, whereas no or little changes were observed in the negative control by qRT-PCR. The gene silencing was further validated by western blot analysis, in which ENO1 protein expression decreased (Fig. 3a).

To determine whether ENO1 was involved in *C. parvum* infection, we treated HCT-8 cells with antibodies against ENO1 and transinfected si-ENO1 to test its effects on the parasite invasion and growth in vitro. Antibodies to ENO1 consistently inhibited the parasite growth by ~ 40–50% in both 3 and 18 h infection assays with *P* values < 0.01 (Fig. 3b). On the other hand, under the effects of siRNA, the infection rate of *C. parvum* was also greatly reduced by ~30–40% in both 3 and 18 h infection assays with *P* values < 0.05 (Fig. 3c).

### ENO1 gene over-expression could increase *C. parvum* infection

To further explore whether ENO1 plays an important role in the invasion of *C. parvum.* We also transfected the expression vector allowing the ENO1 protein to overexpress in HCT-8 cells. The RT-PCR results showed that the parasite load was increased significantly in both 3 and 18 h assays compared with the control culture (*P* < 0.05) (Fig. 3d).

### ENO1 is involved in the degradation of cell membrane proteins during the invasion by *C. parvum*

**Fig. 4:**
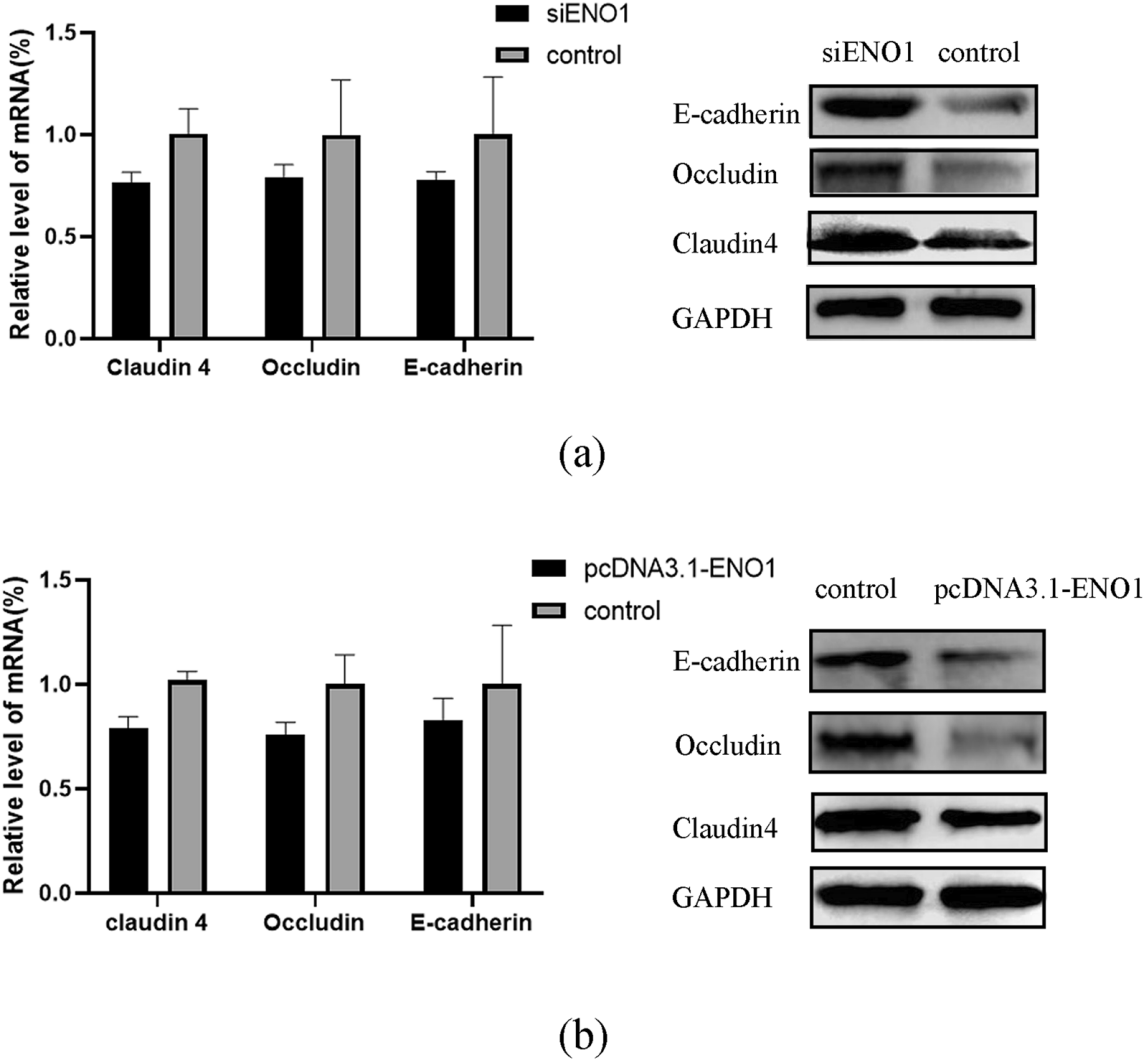
Effects of *Cryptosporidium parvum* infection on the mRNA and protein levels of Occludin, Claudin 4, and E‐cadherin in HCT-8 cells in the presence of siENO1 (**a**) or pcDNA3.1-ENO1 (**b**) transfection

It is worth noting that ENO1, which is located on the surface of the cell membrane, acts as a plasminogen receptor. It is an important component of the fibrinolytic system and can degrade fibrin and extracellular matrix such as laminin and fibronectin [27]. Therefore, to determine whether ENO1 is involved in the degradation of extracellular matrix, we conducted silencing and overexpression experiments of ENO1 protein in HCT-8 cells and studied the effects of *C. parvum* infection on the expression of key proteins that comprise TJ and AJ assembly. The results showed that the mRNA levels of the AJ and TJ proteins (Occludin, Claudin 4, and E‐cadherin) did not change significantly in both silencing and overexpression groups (*P* > 0.05) (Fig. 4a). As shown in Fig. 4b, due to the presence of siENO1, levels of Occluding, Claudin 4, and E-cadherin were higher than control group in varying degrees. On the contrary, the levels of these three proteins are reduced to varying degrees compared with the control group (Fig. 4).

### Sequence and phylogenetic analysis of human ENO1 with other species

**Fig. 5:**
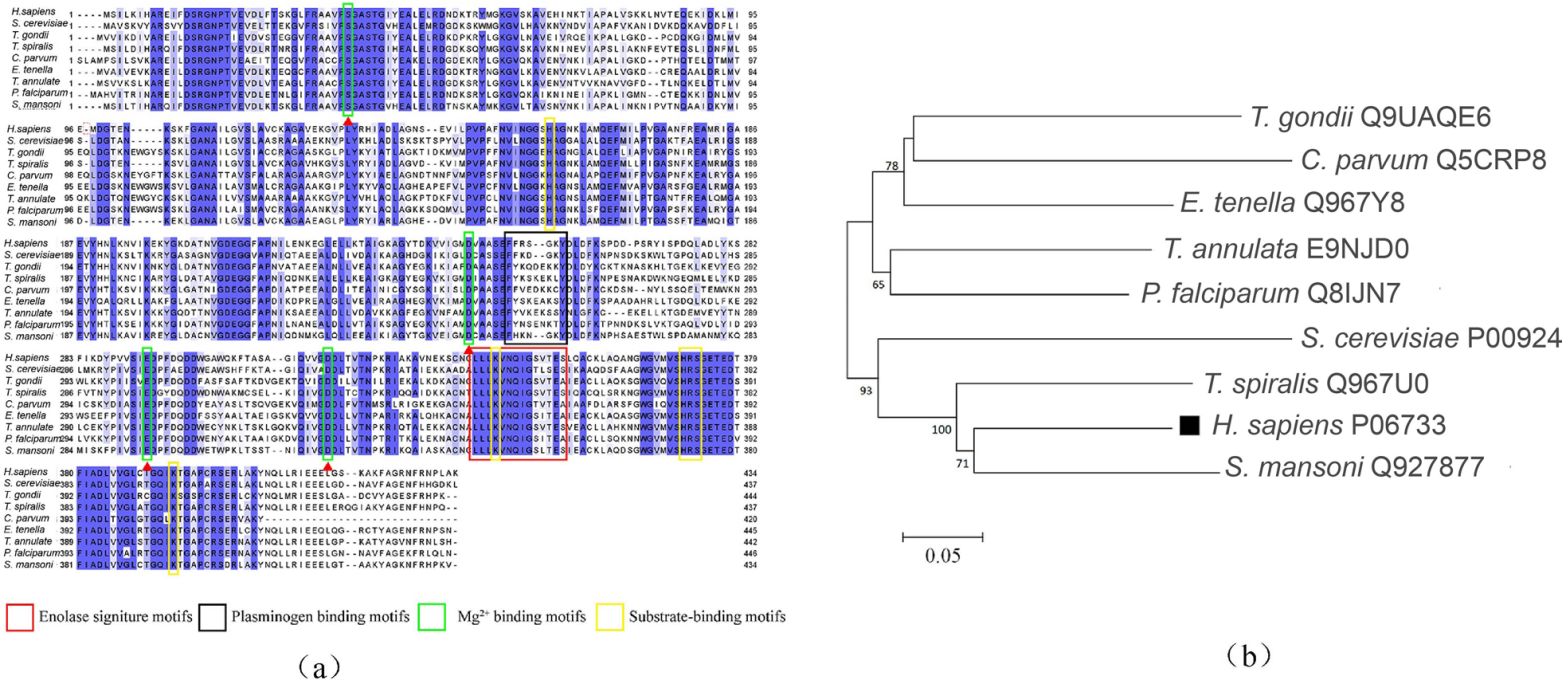
Alignment of amino acid sequence and phylogenetic analysis of human ENO1 with other enolase sequences. **a** Alignment of amino acid sequence of human ENO1 and other species. The Mg2 + binding motifs are shown in a blue box, substrate-binding motifs are shown in a green box, plasminogen binding motifs are shown in a black box, and the enolase signature is shown in red box. The shade of the blue background represents the level of similarity. **b** Phylogenetic analysis of human ENO1 with other enolase sequences. The phylogenetic tree was constructed using the neighbor-joining method. The percentages of replicate trees in which the associated taxa clustered together in the bootstrap test (1000 replicates) are shown next to the branches

The highest homology of amino acid sequences mainly came from apicomplexan protozoa and fungus, such as *S. cerevisiae*, *T. gondii*, *T. spiralis*, *C. parvum*, *E. tenella*, *T. annulate*, *P. falciparum*, and *S. mansoni*, which exhibited 62.90%, 63.26%, 74.07%, 60.79%, 66.59%, 65.89%, 68.21%, and 74.83% similarities, respectively. Using online server analysis, human ENO1 showed a high degree of conservation with other species, including full conservation of the Mg^2+^ binding motifs (S^37^, D^245^, E^293^, and G^317^), substrate-binding motifs (H^158^, E^210^, K^343^, HRS^371−373^ and K^394^). There are certain differences in amino acid sequences in different species at the enolase signature motifs. At the plasminogen binding motif site, the amino acid sequences of each species varied greatly (Fig. 5a). Phylogenetic analysis showed that the human enolase sequence was closest to *S. mansoni* and then other apicomplexan protozoa. *Human sapiens*, Yeas*t*, *T. spiralis*, and *S. mansoni* are located in the same branch (Fig. 5b).

### Truncated ENO1 protein affects the energy supply of HCT-8 cells to *C. parvum*

**Fig. 6:**
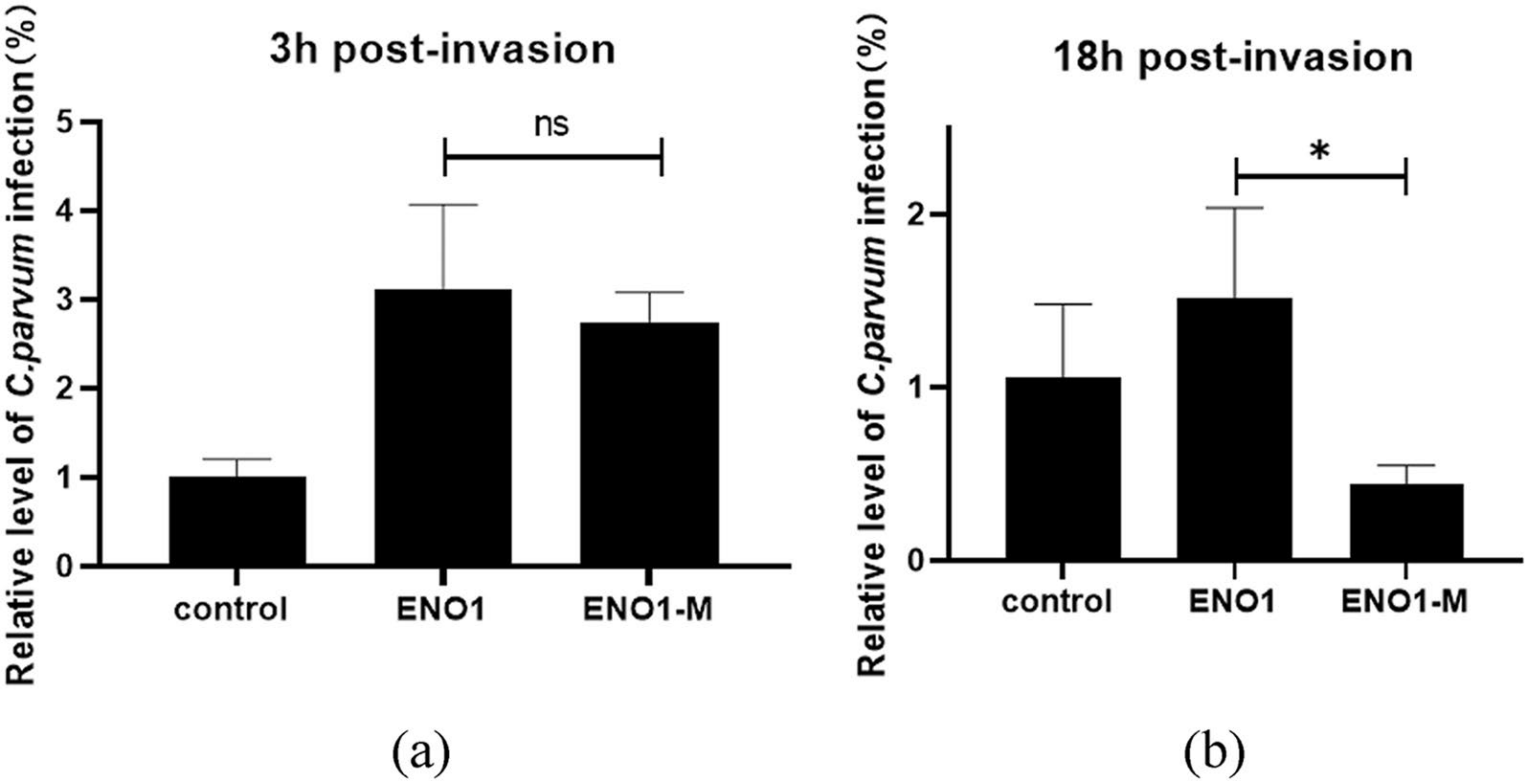
Effect of truncated ENO1 protein on the load of *C. parvum* in HCT-8 cells. **a** 3 h postinvasion and **b** 18 h postinvasion

To investigate the role of the ENO1 protein in *C. parvum* invasion, we constructed a metal ion binding site mutation vector to truncate the ENO1 protein. The results showed that there was no significant difference between these two experimental groups 3 h point in the parasite load, but both were higher than the control group (Fig. 6a). However, at 18 h postinvasion there was a significant decrease in the mutant group compared with the control group (Fig. 6b). The reason for this may be that ENO1 is involved in the glycolysis process, and the mutant protein affects the pathways related to glycolysis, which in turn affects the developmental stage of *C. parvum* in cells.

## Discussion

The life cycle of *C. parvum* includes the stages of sporozoite adhesion and invasion into the host cell, intracellular development, and reproduction. Among them, the invasion of the host cell by sporozoites after decapsulation is the first step to establish the infection [28]. It has been reported that protein interactions between sporozoites and host cells are main players mediating parasite invasion and intracellular progression in host cells, and Cpgp40 is one of the key proteins with biological importance. The surface location of Cpgp40 has been clearly demonstrated and functional studies of this protein proved that it can interact with host cell proteins to facilitate the invasion of sporozoites [17, 25]. Therefore, identification of molecules interacting with Cpgp40 would be a key step to understand the molecular mechanisms of parasite invasion mediated by this parasitic protein.

In the present study, we obtained 14 proteins that have potential interactions with Cpgp40. By constructing the fusion protein GST-Cpgp40 as a probe, proteins derived from HCT-8 cells were incubated and initially analyzed for screening of candidate proteins potentially interacting with Cpgp40. Proteins binding with GST only were eliminated through setting up the GST control, and the remaining proteins were determined as candidate receptors for Cpgp40. Bioinformatics analysis revealed a wide subcellular distribution for the candidate proteins, as presented in Additional file 5: Table S4. Proteins located on the cell membrane and cytoskeleton are of particular interest, including alpha-enolase (ENO1), protein S100-A8 (S100/A8), immunoglobulin heavy constant gamma 3 (IGHG3), actin, tubulin beta chain (TUBB), and tubulin alpha-3D chain (TUBA3D). While intestinal epithelial cells are the main target for *C. parvum* invasion [29], their functional impairment caused by the destruction of the extracellular matrix and the connection complex on the cell membrane, such as some tight junctions (TJs) and adherent junctions (AJs) [30–32], would facilitate the invasion of *C. parvum.* For the identified proteins potentially interacting with Cpgp40, cell membrane and cytoskeleton related proteins, such as ENO1, actin, TUBB, and TUBA3D, have been reported to be associated with the functionality of tight junctions and adherent junctions. Therefore, these proteins were the main targets for further verification of their interactions with Cpgp40.

Expression profiles revealed significant downregulation of mRNA for proteins ENO1, ACTG1, TUBB, and TUBA3D at 3, 6, and 12 h post parasite infection, respectively (Fig. 1). Proteins ACTG1, TUBB, and TUBA3D are all components of the cytoskeleton, involved in cellular structural support, intracellular transport and DNA isolation. Downregulation of these proteins agrees with their functional activity during parasite invasion, such as actin polymerization and tight junction disruption [33–36]. Unlike proteins of cytoskeleton, ENO1 is distributed both on cell membrane and in cytoplasm. Significant downregulation of ENO1 mRNA at 3 h post *C. parvum* infection indicated the involvement of this protein during parasite invasion. Moreover, bioinformatics analysis of ENO1 presented the following reasons for selecting this protein as the main candidate receptor for Cpgp40. Initially, the protein size for ENO1 is about 43 kDa, which is in line with the silver staining results. Moreover, studies of ENO1 from parasites, such as *Giardia intestinalis* and *Trichinella spiralis*, have proved the participation of ENO1 during parasite invasion [37, 38]. It is worth noting that ENO1, which is located on the surface of the cell membrane, functions as a plasminogen receptor. Plasminogen is the precursor of plasmin, an important component of the fibrinolytic system, and can degrade fibrin, adhesion protein and fibronectin [27]. In the process of many parasites infecting the host, it is mainly to use the host’s own fibrinolytic system to break through the tissue barrier and accelerate the infection [39]. Through forming a silent complex with urokinase receptors, integrins, and cytoskeleton proteins, ENO1 perform the function of plasminogen receptors and degrade the extracellular matrix [40, 41]. Therefore, we preliminarily believe that the ENO1 protein plays a role in the invasion of intestinal epithelial cells by *C. parvum*. We mainly used CO-IP and BiFC experiments to verify the interaction between *C. parvum* gp40 and ENO1. These experiments all showed that the two proteins had solid interaction (Fig. 2).

These experiments all showed that there was indeed the possibility of interaction between them. While we have made some attempts in the early time to obtain the crystal structure of Cpgp40 but failed due to its glycoprotein secretion and unstable structure. And because of the lack of crystal structure model, the predicted Cpgp40 structures are extremely variable.

The participation of ENO1 during *C. parvum* invasion was then studied through gene manipulation. By using the designed siRNA, mRNA of ENO1 was silenced in HCT-8 cells, resulting in decreased expression of ENO1. The infection rates of *C. parvum* in these cells showed significant reduction with *P*-values < 0.01 both at 3 and 18 h postinfection, which were consistent with the reduced infection rates caused by antibody neutralization. Conversely, the increased expression of ENO1 protein promoted the parasite infection. These results illustrated that ENO1 was involved in *C. parvum* infection, conducive to the parasite invasion and may serve as a key enzyme for parasite progression (Figs. 1 and 3).

As shown in the previous observation, the mRNA level of ENO1 in HCT-8 cells decreased at 3 h post *C. parvum* infection and returned to the normal level at 6 and 12 h postinfection (Fig. 1). Considering its functional facilitation of parasite invasion observed through gene manipulation, we speculated that at the early stage of invasion, with the massive invasion of parasites, the cell membrane surface protein dissolved more to form a negative feedback system, which led to a decrease in the transcription level of the target protein gene. Given the brief duration of parasite invasion, developmental stages transition to the trophozoite stage within 2 h postinfection [42]. Sugar and nucleotide transporters are upregulated as the parasite starts to acquire nutrition from the host [43]. Furthermore, ENO1 is an important glycolytic enzyme that can catalyze the mutual conversion of 2-phosphoglycerate and phosphoenolpyruvate, which maintain intracellular ATP levels [44]. Therefore, the back to normal expression of ENO1 would be explained to maintain energy requirement for parasite progression inside host cells.

Protein interaction and gene manipulation proved the involvement of ENO1 during *C. parvum* infection. We subsequently investigated the possible functional mechanisms of ENO1 during parasite invasion and in vivo development. Under the conditions of *ENO1* gene silencing and overexpression, mRNA and protein expression levels of human Occludin, Claudin 4, and E‐cadherin were examined to verify whether ENO1 is involved in the degradation of tight junction (TJ) and adhesion junction (AJ) proteins related to the extracellular matrix. The results showed that in the presence of *ENO1* gene silencing, the protein levels of the three proteins were significantly higher than those of the control group, although mRNA levels of these proteins remained unchanged (Fig. 3). These results suggested that during parasite invasion, gp40 and ENO1 may interact to regulate the expression of TJ and AJ proteins, which can influence the permeability of cell membrane for parasite invasion.

Multiple protein sequence alignment of ENO1 with sequences derived from some species of apicomplexan parasites revealed the similarities between them were from 60% to 75%. However, the homology of amino acids at the main functional sites were 100%, indicating the conservation of functionality for ENO1. Therefore, we mutated the ENO1 metal ion binding sites, which are mainly related to the enzyme function of ENO1, to detect whether glycolycolysis-related processes affect the parasite load of cells. The results showed that the parasite load decreased significantly after 18 h postinfection, but was not influenced at 3 h postinfection, suggesting that the enzyme function of ENO1 did affect the endogenous development of *C. parvum* but had almost no effect at the initial stage of invasion of *C. parvum*.

This study identifies a key host cell protein ENO1 that can directly interact with Cpgp40. Functional analysis of ENO1 revealed its participation during both parasite invasion and endogenous development. Of course, subsequent studies on the structure of ENO1–gp40 protein complexes is particularly important. Crystal studies of these two proteins will help to understand the mechanism of protein interaction and further develop anticryptosporidium drugs and vaccines. However, due to *C. parvum* gp40 protein is secreted protein, it is very difficult to obtain its crystal structure. In addition, the related pathways involved in ENO1 will be required to fully understand the specific role and function mechanism of ENO1 in the parasite infection of host cells.

## Conclusions

In this study, we utilize the purified Cpgp40 as a bait to obtain host cell proteins interacting with Cpgp40 through GST pull-down method and identified the alpha-enolase protein from HCT-8 cells, which showed direct interaction with Cpgp40. Functional studies illustrated that the host cell protein ENO1 was involved in the regulation of tight junction and adherent junction proteins during *C. parvum* invasion and was required for endogenous development of *C. parvum.* Further studies are needed to fully understand the specific role and functional mechanism of ENO1 in the invasion of the host by *C. parvum*.

### Supplementary Information

**Additional file 1. **The LC–MS/MS protocol.

**Additional file 2: Table S1.** Primers used in this study. **Table S2.** Plasmids used in this study. **Table S3.** Gene‐specific primers used for real‐time PCR analysis of mRNA levels.

**Additional file 3: Figure S1.** Silver-stained one-dimensional SDS-PAGE of GST-fusion protein coupled beads (GST-Cpgp40).

**Additional file 4: Figure S2.** The Venn diagram of the differentiated protein obtained by LC–MS identification.

**Additional file 5: Table S4.** The protein summary obtained by LM/MS.

**Additional file 6. **The raw materials obtained by LM/MS.

## Data Availability

The LS/MS sequencing data referenced in this paper can be accessed through Additional files 5 and 6.

## Abbreviations

HEK239cells: Human embryonic kidney 293 cells
HCT-8 cells: HCT-8 human ileocecal adenocarcinoma cells
*C. parvum*: *Cryptosporidium parvum*
siRNA: Short-interfering RNA
PBS: Phosphate-buffered saline
AJs: Adherent junctions
TJs: Tight junctions
