# Supplementary material for: Identification of host protein ENO1 (alpha-enolase) interacting with Cryptosporidium parvum sporozoite surface protein, Cpgp40
Source: Parasit Vectors. 2024 Mar 19;17:146. doi: 10.1186/s13071-024-06233-5 (PMC10953254; doi:10.1186/s13071-024-06233-5)
Supplement: Supplementary file 1 — Additional file 1. The LC–MS/MS protocol. [file 13071_2024_6233_MOESM1_ESM.pdf]

LC-MS/MS protocol

The final concentration of 10 mm dithuleneol (DTT) was added to the sample to reduce the protein, followed by the final concentration of 55 mM iodide acetylamide (IAM), and finally to the trypsin enzyme of 1 μg, which was used overnight to digest 8 h to 16 h. The polypeptides produced by enzymatic solution are desalinated with C18 columns, and the desalinated peptides are drained and dissolved with 15 μl Buffer (0.1% acetic acid, 3% acetylene). The samples were analyzed with LC-MS/MS (ekspertTMnanoLC; AB Sciex TripleTOF 5600-plus) according to standard protocols. For IDA (Information Dependent Acquisition), the MS spectrum is scanned with an ion accumulation time of 250 ms, and the MS spectrum of 30 precursor ions is acquired with an ion accumulation time of 50 ms. Set the precursor ion dynamic exclusion time to 15 s. And then Submit the original down-machine data directly to the Proteinpilot for database retrieval. For protein identification, the Paragon algorithm within ProteinPilot was employed to interrogate the UniProt Human Database. Peptides with an unused score greater than 1.3, indicating a confidence level exceeding 95%, were classified as credible peptides. Proteins harboring at least one unique peptide were subsequently retained in the analysis. UniProt (https://www.uniprot.org/) was used to search the subcellular localization and structure identified protein.
