# Supplementary material for: Identification of host protein ENO1 (alpha-enolase) interacting with Cryptosporidium parvum sporozoite surface protein, Cpgp40
Source: Parasit Vectors. 2024 Mar 19;17:146. doi: 10.1186/s13071-024-06233-5 (PMC10953254; doi:10.1186/s13071-024-06233-5)
Supplement: Supplementary file 2 — Additional file 2: Table S1. Primers used in this study. Table S2. Plasmids used in this study. Table S3. Gene‐specific primers used for real‐time PCR analysis of mRNA levels. [file 13071_2024_6233_MOESM2_ESM.pdf]

Table S1. Primers used in this study

Primer name	Sequence[1](5' to 3')	Restriction enzyme sites
pGEX-4T-1-Cpgp40-F	CGC*GAATTC*GATGTTCCTGTTGAGGGCTC	*EcoRI*
pGEX-4T-1-Cpgp40-R	CGC*GTCGAC*CTCTGAGAGTGATCTTCTTG	*SalI*
pcDNA3.1-ENO1-F	CGC*GAATT*CGCCACCATGTCTATTCTCAAGATCCATGCC	*EcoRI*
pcDNA3.1-ENO1-R	CCG*CTCGAG*TTACTTGGCCAAGGG	*XhoI*
pBiFC-VN155-ENO1-F	CGC*GAATT*CGCCACCATGTCTATTCTCAAGATCCATGCC	*EcoRI*
pBiFC-VN155-ENO1-R	CCG*CTCGAGC*TTACTTGGCCAAGGG	*XhoI*
pcDNA3.1-HA-Cpgp40-F	CGC*GAATTC*GCCACCGATGTTCCTGTTGAGGGCTC	*EcoRI*
pcDNA3.1-HA-Cpgp40-R	CGC*GTCGAC*GTGGTGGTGGTGGTGGTGCTCTGAGAGTGATCTTCTTG	*XhoI*
pBiFC-VC155-HA-Cpgp40-F	CGC*CTCGAG*GCCACCGATGTTCCTGTTGAGGGCTC	*EcoRI*
pBiFC-VC155-HA-Cpgp40-R	CGC*GTCGAC*GTGGTGGTGGTGGTGGTGCTCTGAGAGTGATCTTCTTG	*XhoI*
pCAGGS-HA-ENO1-F	CGC*GAATT*CGCCACCATGTCTATTCTCAAGATCCATGCC	*EcoRI*
pCAGGS-HA-ENO1-R	CCG*CTCGAG*TTACTTGGCCAAGGG	*XhoI*
pCAGGS-HA-ENO1-M1-F	CCGCTGGTGCTTCAACTGGTATCTATGAGG	/
pCAGGS-HA-ENO1-M1-R	AGTTGAAGCACCAGCGGGCACAGCAGCTCTGAAGA	/
pCAGGS-HA-ENO1-M2-F	TCATCGGCATGGCCGTAGCGGCCTCCGAGTTCTT	/
pCAGGS-HA-ENO1-M2-R	TACGGCCATGCCGATGACCACCTTATC	/
pCAGGS-HA-ENO1-M3-F	GTCTATCGCAGATCCCTTTGACCAGGATGACTG	/
pCAGGS-HA-ENO1-M3-R	AGGGATCTGCGATAGACACCACTGGGTAGTCCTTG	/
pCAGGS-HA-ENO1-M4-F	AGGTAGTGGCGGATGATCTCACAGTGACCAACCC	/
pCAGGS-HA-ENO1-M4-R	ATCATCCGCCACTACCTGGATTCCTGCACTGG	/

[1] Restriction enzyme sites are presented in italics.

Table S2. Plasmids used in this study.

Plasmids	Used for
pGEX-4T-1-Cpgp40	Prokaryotic express to produce GST-Cpgp40 fusion protein for GST Pull-down
pcDNA3.1-ENO1	Eukaryotic express to produce ENO1 fusion protein for Co-IP
pcDNA3.1-HA-Cpgp40	Eukaryotic express to produce HA-Cpgp40 fusion protein for Co-IP
pBiFC-VC155-HA-Cpgp40	Eukaryotic express to produce HA-Cpgp40 fusion protein for BiFC
pBiFC-VN155-ENO1	Eukaryotic express to produce HA-KN151 fusion protein for BiFC
pCAGGS-HA-ENO1	Eukaryotic express to study the effect of ENO1 in *C. parvum* invasion HCT-8 cells
pCAGGS-HA-ENO1-M	Eukaryotic express truncated ENO1 protein

Table S3. Gene specific primers used for real time PCR analysis of mRNA levels

Gene	Primer sequence (5′-3′)
Human ENO1	F: GGAGATCTCGCCGGCTTTAC
	R: TCAACAGCCTTTGAGACACCCTT
Human GAPDH	F: GTCAGCCGCATCTTCTTTTG
	R: GCGCCCAATACGACCAAATC
Cryptosporidium parvum 18S	F: TAGAGATTGGAGGTTGTTCCT
	R: TAGGGTAGGCACACGCTGAGCC
Human 18s	F: CCGATAACGAACGAGACTCTGG
	R: TAGGGTAGGCACACGCTGAGCC
Human occludin	F: CCCCATCTGACTATGTGGAAAGA
	R: AAAACCGCTTGTCATTCACTTTG
Human claudin 4	F: GGCGTGGTGTTCCTGTTG
	R: AGCGGATTGTAGAAGTCTTGG
Human E-cadherin	F: ATTTTTCCCTCGACACCCGAT
	R: TCCCAGGCGTAGACCAAGA
Human ACTG1	F: CATTGTCATGGACTCTGGAGAC
	R: GAGGATCTTCATGAGGTAGTCG
Human TUBB	F: GCAATAGCACAGCCATCCAGGAG
	R: TCAGCCTCGGTGAACTCCATCTC
Human TUBA3D	F: GCTCTCAGTGGATTACGGCAAGAAG
	R: TGGGTGGTCAGGATGGAGTTGTAG
