# Supplementary figures and images for: Identification of host protein ENO1 (alpha-enolase) interacting with Cryptosporidium parvum sporozoite surface protein, Cpgp40
Source: Parasit Vectors. 2024 Mar 19;17:146. doi: 10.1186/s13071-024-06233-5 (PMC10953254; doi:10.1186/s13071-024-06233-5)

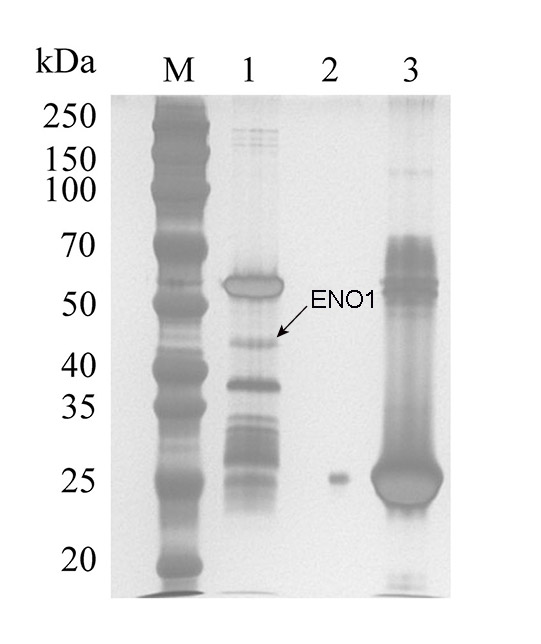

Supplement: Supplementary file 3 — Additional file 3: Figure S1. Silver-stained one-dimensional SDS-PAGE of GST-fusion protein coupled beads (GST-Cpgp40). [file 13071_2024_6233_MOESM3_ESM.jpg]

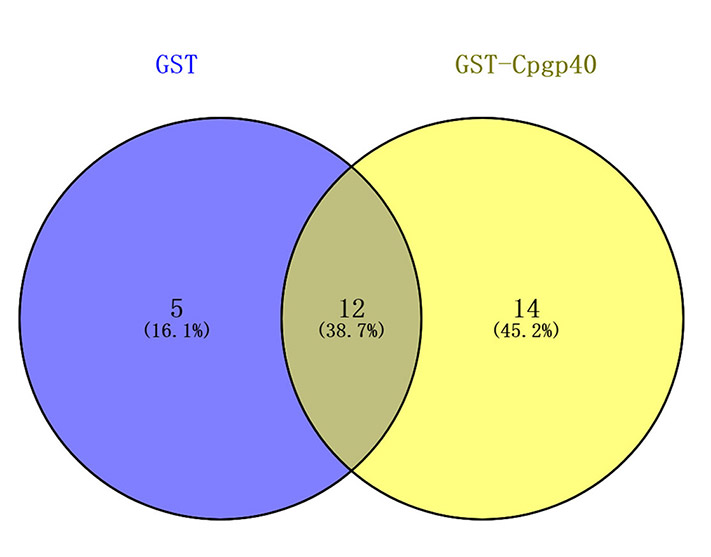

Supplement: Supplementary file 4 — Additional file 4: Figure S2. The Venn diagram of the differentiated protein obtained by LC–MS identification. [file 13071_2024_6233_MOESM4_ESM.jpg]
